# The Ensembl Regulatory Build

**DOI:** 10.1186/s13059-015-0621-5

**Published:** 2015-03-24

**Authors:** Daniel R Zerbino, Steven P Wilder, Nathan Johnson, Thomas Juettemann, Paul R Flicek

**Affiliations:** European Molecular Biology Laboratory, European Bioinformatics Institute, Wellcome Trust Genome Campus, Hinxton, Cambridge, CB10 1SD UK

## Abstract

Most genomic variants associated with phenotypic traits or disease do not fall within gene coding regions, but in regulatory regions, rendering their interpretation difficult. We collected public data on epigenetic marks and transcription factor binding in human cell types and used it to construct an intuitive summary of regulatory regions in the human genome. We verified it against independent assays for sensitivity. The Ensembl Regulatory Build will be progressively enriched when more data is made available. It is freely available on the Ensembl browser, from the Ensembl Regulation MySQL database server and in a dedicated track hub.

## Background

Despite our increasing knowledge of genomes and their variants, the downstream effects of sequence variants and the affected cellular mechanisms are still poorly understood. In particular, a large number of the variants identified in genome-wide association studies are located in non-protein coding regions [1], and are presumed to affect gene expression regulation. Similarly, it has been proposed that a significant fraction of the potential for phenotypic adaptation lies within the regulatory elements of the genome [2,3].

There is still much to learn about the dynamic regulation of gene expression [3,4]. *Cis*-regulatory elements are short segments of the genome that either recruit transcription factors (TFs) or affect the properties of the messenger RNA as it is being transcribed [5]. Gene expression is also highly tied to transmissible epigenetic marks [6-8]. The DNA molecule and the histone proteins it is wrapped around can be modified with various additions, such as methyl, acetyl or phosphate groups. These alterations have been shown to provide crucial markers of developmental diseases [9] and cancer [10]. Finally, the three-dimensional conformation of the DNA molecule also affects its activity. In particular, it determines which regions are accessible to outside molecules [11], and which regions are in physical proximity to each other despite being distant in the genomic sequence [12].

Various experimental techniques help us identify the epigenetic markers of the genome and the putative underlying *cis*-regulatory elements. Chromatin immuno-precipitation (ChIP) coupled with either genome-wide tiling microarrays (ChIP-chip [13]) or direct high-throughput sequencing (ChIP-Seq [14-16]) make it possible to perform genome-wide and protein-specific measurements of DNA binding, as well as detect a range of histone modifications. Other methodologies have been developed to identify modified cytosine bases, ranging from array-based approaches such as MeDIP-chip [17], through to more exhaustive approaches such as whole-genome bisulphite sequencing [18]. Regions of open chromatin can be mapped using formaldehyde-assisted isolation of regulatory elements (FAIRE) [19], nuclease digestion by DNase1 coupled with high-throughput sequencing (DNase-seq) [20] or assaying transposase-accessible chromatin (ATAC-seq) [21].

Significant efforts to provide genome-wide maps of histone modifications have already proved successful in elucidating some of the basic patterns associated with promoter and enhancer regions [14,15,22,23]. In addition to an explosion of small and medium-scale studies producing this type of data, large-scale projects like ENCODE [24,25], Roadmap Epigenomics [26], and Blueprint [27] are releasing large amounts of valuable data into the public domain. With the promise of even higher sequencing throughput, genome-wide epigenomic datasets will only become more abundant.

One important challenge is to bring together and standardize these studies, in order to integrate all the information into a coordinated regulatory annotation of the genome. To address this challenge we developed the Ensembl Regulatory Build, within the Ensembl project [28], to provide a high-level overview of the regulatory activity of the genome. Through this process, we annotate putative regulatory regions from public experimental data, and associate these regions with regulatory function.

## Results

**Figure 1:**
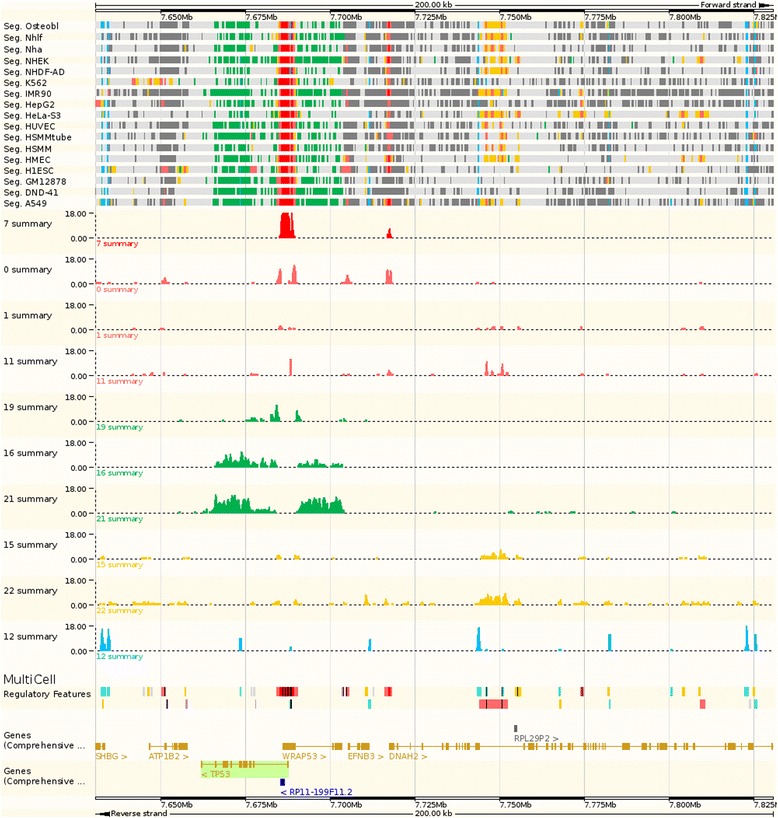
**The Regulatory Build process.** In a first step we run segmentation software across multiple cell types. For each cell type and at each base pair, the genome is assigned a state, identified by an arbitrary number assigned by the segmentation software. We assign to each state a non-unique functional label, represented by its color on the browser, as shown at the top. For each state at each base pair, we compute the number of cell types sharing that state at that position, as shown in the center of the figure. Having selected relevant states and set some thresholds, we define regions of interest, which are the foundation of the regulatory build. These regions are then complemented with unannotated ChIP-Seq transcription factor binding site peaks and unannotated DNase1 hypersensitivity sites.

We defined genomic regions of interest characterised by biochemical activity through a four-step Regulatory Build process that combined all available data, summarised in Figure 1.

We first reduced all the experimental data for each cell type into a cell type-specific annotation of the genome. This can be done with segmentation tools, such as Segway [29] or ChromHMM [30]. In a first training pass, these algorithms take as input a set of genome-wide assays, and detect recurring signal patterns (referred to as 'states'). In a segmentation pass, for each cell type at each base pair of the genome, they determine the most likely underlying state, based on local experimental measurements.

**Figure 2:**
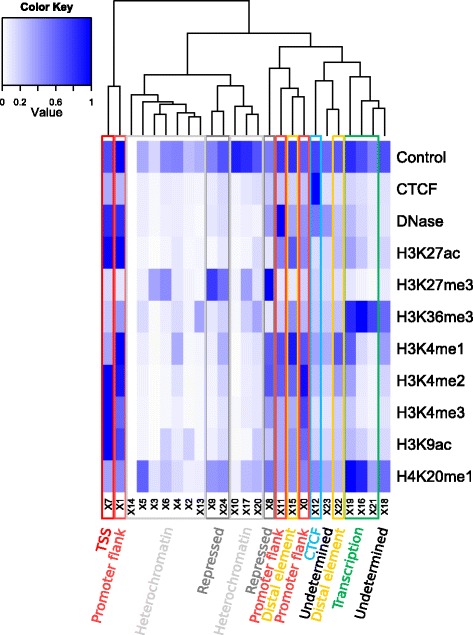
**Experimental marks associated with different labels.** This heatmap represents the experimental marks and the label associated with each state. The states were defined by Segway, and the labels assigned by the Ensembl Regulatory Build *a posteriori*. Although the label assignment relies mainly on overlaps with known features, the states with the same labels co-cluster based on their experimental marks. The main exception are the promoter flanking states, which cluster either with promoters or with distal *cis*-regulatory elements. In effect, these states tend to represent a mixture of the other two.

By overlapping these segmentation states, produced by unsupervised machine learning, with known genomic features, we assigned them functional labels, such as ‘predicted promoter with TSS’ (where TSS is transcription start site), ‘predicted transcribed region’, ‘predicted promoter flank’, ‘predicted enhancer’, ‘CTCF enriched’, ‘predicted repressed’, ‘predicted low activity’, ‘predicted heterochromatin’. To ensure the broadest applicability of our approach, we minimized the use of known epigenetic marks when assigning labels, rather using prior annotations. We nonetheless verified after the fact that states with similar labels display similar histone marks, as shown in Figure 2.

We then defined consensus regions of interest, referred to as ‘MultiCell’ regulatory features. To do so, for each of the labels ‘predicted promoter with TSS’, ‘predicted promoter flank’, ‘predicted enhancer’ and ‘CTCF enriched’, we computed a summary function, which represents at any given base pair how many cell types have one of the corresponding segmentation states. We then computed contiguous regions where this summary function is above a threshold, set to optimally fit the global TF binding signal (see Materials and methods section). In addition to these regions, we added regions where TF binding or open chromatin were reported, yet were not covered by the previous annotations.

Finally, the MultiCell features defined above were annotated with cell type-specific activity levels. This activity level was obtained by querying, for each feature, the presence or absence of cell type-specific evidence associated with that feature’s label.

We examined the properties of the consensus annotation, as shown in Table 1. The overall coverage of the genome is 12.9%, which is commensurate with previous estimates [25]. The promoters, including attached flanking regions, are by far the largest elements (mean length 4.4 kb), whereas distal enhancers and CTCF binding sites are shorter (respectively 547 and 622 bp on average), but far more numerous (respectively 127,786 and 117,711 elements). Finally, proximal enhancers, defined as flanking regions detached from any promoter, cover the greatest number of bases (160 Mbp in total).

**Table 1:** **Summary details for the regulatory build in Ensembl release 76**

**Type**	**Number**	**Average length (bp)**	**Standard deviation (bp)**	**Total length (Mbp)**	**Genome coverage (%)**
Promoters	16,488	4,369	2,746	72	2.3%
Proximal enhancers	85,526	1,876	1,741	160	5.2%
Distal enhancers	127,786	547	482	70	2.3%
CTCF binding	117,711	622	1,206	73	2.4%
Unannotated transcription factor binding site	27,523	528	628	15	0.5%
Unannotated open chromatin	71,568	502	346	36	1.2%
Total	446,602			399	12.9%

To corroborate our annotation, we compared it with other reference annotations. Of the 217,516 strict TSS calls found with CAGE tags by the FANTOM 5 consortium [31], 88.9% were recovered. Of the 882 validated human VISTA enhancers [32], 92.4% were recalled in our build. Finally, 80.3% of the 38,533 robust enhancers called by FANTOM 5 [33] were covered by one of our annotations.

## Discussion

By design, this annotation of the genome is focused on the pragmatic need to define epigenomic markers across samples. Its regulatory features are phenomenological, that is, defined by biochemical signal alone [34]. If only because of the resolution of epigenetic marks (generally at nucleosome scale), they are probably a broad extension of the biochemically active bases in the genome. At the same time, we focused exclusively on the marks associated with transcriptional regulation. This compromise led us to annotating 12.9% of the human genome.

A key parameter that can distort the segmentation is the number of states used by the machine-learning algorithm. Instead of trying to optimize the number of states, we circumvented this issue by focusing on the biologically meaningful labels that are ultimately provided to the user. There are only eight such labels, and Figure 2 illustrates that nearly all labels have more than one underlying state. This suggests that the granularity of the segmentation was sufficient for our purpose, that is, distinguishing these eight labels.

The build process reduces inherently noisy and complex biological data into a tidy and easy to understand summary. Consequently, subtle patterns can be masked from the user. To mitigate this loss of information, all the data used in the Regulatory Build, namely the experimental signal and the segmentations, are available through the Ensembl Browser.

The Ensembl Regulatory Build is by no means a final product, rather a continuing process that will be extended and enriched in the coming years. In future Ensembl releases, we will be importing more and more datasets, covering more cell types, as they are made available. This will provide greater sensitivity to detect transient elements that are only active in a few cell types. Also, we are starting to receive normal cell and tissue data, as opposed to cell lines. Coupled with knowledge of cell differentiation pathways, these data will help illuminate the key epigenomic marks associated with cell fate.

We will also be refining our annotation of regulatory features. The architecture of the Ensembl Regulatory Build process will allow us to take full advantage of ongoing research in machine learning, and genome segmentation in particular. We hope to extend the vocabulary used to describe the elements and the activity levels. For example, we wish to distinguish poised, repressed and closed elements, instead of applying a binary active/inactive notation.

The remaining open question is how to confidently assign gene targets to *cis*-regulatory elements. A number of experimental assays are being investigated, such as statistical correlation [35], chromatin conformation assays [36-38] or expression quantitative trait loci studies [39,40]. The Ensembl framework, which currently holds a consistent relation database of gene transcripts [41], variants [42], and now regulatory elements will be a natural home for this key component of cell biology.

## Conclusions

The Ensembl Regulatory Build aims to provide the most up-to-date and comprehensive survey of the regulatory elements of the genome, in the same way the Ensembl Genebuild maintains a reliable summary of known gene sequences. Centralizing datasets from various large-scale projects, we process them with a uniform pipeline, then compute an exhaustive and robust annotation of the regulatory elements of the genome. Although this annotation will likely evolve in the years to come, the regions are already assigned stable identifiers, providing a solid framework for ongoing epigenomic research.

## Materials and methods

### Source data

We chose to run our segmentation (see below) on a pre-selected set of ChIP-Seq assays (CTCF, H3K4me1, H3K4me2, H3K4me3, H3K9ac, H3K27ac, H3K27me3, H3K36me3, H4K20me1) along with DNaseI hypersensitivity and a control ChIP-Seq experiment. We therefore downloaded from ENCODE 2 and Epigenomics Roadmap all the raw read datasets produced by ChIP-Seq and DNaseI hypersensitivity experiments on the 18 cell types that had all of the above required assays: A549, DND-41, GM12878, H1-hESC, HeLa-S3, HepG2, HMEC, HSMM, HSMMtube, HUVEC, IMR90, K562, Monocytes-CD14+, NH-A, NHDF-AD, NHEK, NHLF, Osteoblast. Including replicates and control samples, this amounted to 740 datasets, all referenced in the Ensembl homo_sapiens_funcgen_76_38 MySQL database.

### Uniform processing of sequencing data

Most studies using epigenomic data present their own analysis and results, which often differ from each other in small, but relevant details. In the current absence of standardized practices, and to make all data as homogeneous as possible, raw sequencing reads from these experiments were processed with a uniform in-house analysis pipeline.

For each ChIP-Seq experiment, the raw sequencing reads were mapped to the GRCh38 human genome assembly using bwa samse [43] with default parameters.

We called punctate peaks using SWEMBL [44]. We filtered SWEMBL peaks on their score, using a fixed permissive threshold (-f 150 -R 0.0005 -d 150), then retained the highest scoring peaks, as defined by the ENCODE Irreproducibility Discovery Rate (IDR) process [45] with an IDR threshold of 0.01 for datasets with more than 100,000, and 0.05 for smaller datasets, as recommended by the IDR developers. To account for large differences in the number of reads between replicates, the number of retained peaks was scaled linearly to half the ratio between the largest and smallest estimated numbers of peaks.

To detect broader regions, such as H3K36me3 and H3K27me3 enrichment, we used CCAT [46]. We filtered out peaks falling within known problematic regions, defined on GRCh38 using the same process as the Duke ENCODE excluded regions [47].

### Genome segmentation

The coverage signal was normalized within each dataset using align2rawsignal [48], with options (-w = 180 -n = 5). The segmentation was run across all the resulting datasets using Segway, with options (--num-labels = 25 --num-instances = 10 --resolution = 200 --prior-strength = 1000 --ruler-scale = 200 -m 1,2,3,4,5,6,7,8,9,10,11,12). For performance reasons, training was only computed on the ENCODE pilot regions. The segmentations were masked across the same problematic regions as the peaks.

### Computing transcription factor binding densities

For each TF *t*, we computed a summary function *p*_*t*_ across the genome representing the number of overlapping peak calls at that position divided by the number of assays. This function represents an approximate binomial estimator for the existence of a peak across the observed experiments.

We then computed an overall TF binding probability function assuming approximate independence between the binding probabilities of the different transcription factors:

$$ {p}_{TF}=1-{\displaystyle \prod_{t\in TF}}\left(1-{p}_t\right) $$

### Assigning labels to segmentation states

**Figure 3:**
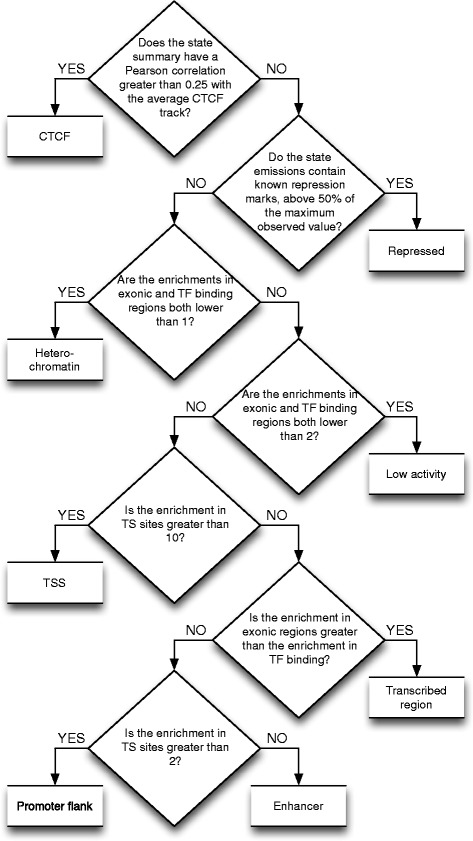
**Decision tree assigning labels to unsupervised segmentation states.**

For each segmentation state *s* we constructed a summary function *f*_*s*_ representing for each base pair the number of cell types that are in state *s* at that position. We computed the enrichment of contiguous regions where *f*_*s*_ was strictly positive for TF binding, TSSs and exonic regions. We also computed the Pearson correlation of *f*_*s*_ to the CTCF density. The state *s* was then assigned a label using the decision tree represented in Figure 3.

### Defining regions of interest through cutoff optimization

We assume that the labels we are interested in, namely *cis*-regulatory elements, promoters and insulators, are correlated to TF binding. Given a cutoff *k* we computed the enrichment for TF binding signal p_TF_ of regions where *f*_*s*_ was strictly greater than *k*. If we found a value of *k* such that this enrichment was greater than 2, then the segmentation state was retained for the next step.

For each label *l*, we then set a cutoff *k*_*l*_ that maximized the F-score *F*_*l,k*_ where *S*_*l*_ is the set of states which were assigned that label and passed the above test, and:

$$ \begin{array}{l}{f}_l={\displaystyle \sum_{s\in {S}_l}}{f}_s\\ {}{\delta}_{l,k}=\left\{\begin{array}{c}\hfill 1\  if\ {f}_l>k\hfill \\ {}\hfill 0\  otherwise\hfill \end{array}\right.\\ {}Se=\frac{{\displaystyle \int }{p}_{TF}.{\updelta}_{l,k}}{{\displaystyle \int }{p}_{TF}}\\ {}Sp=\frac{{\displaystyle \int }{p}_{TF}.{\updelta}_{l,k}}{{\displaystyle \int }{\updelta}_{l,k}}\\ {}{F}_{l,k}=2\frac{Se.Sp}{Se+Sp}\end{array} $$

Having computed *k*, we computed the contiguous regions where *f*_*l*_ was greater than *k*_*l*_.

For simplicity, enhancer elements that overlapped promoter flanks were merged into the latter. Promoter-flanking regions that overlapped promoters were merged into the flanks of the promoter element. Because of their structural significance, CTCF binding sites were not merged into overlapping elements.

If any contiguous regions where *p*_*TF*_ was greater than 0 did not overlap one of the segmentation-based annotations defined above, it was added into the Build, marked as ‘TF binding site’.

Finally, we computed the overlap of all observed open chromatin regions. If one of those did not overlap any of the annotations defined above, it was added into the Build, labeled as ‘Open Chromatin’.

### Determining cell-specific activity

We then annotated the activity of these features in each cell type with a binary active/inactive label. For each region defined by segmentation data, we searched for an overlap in that cell type’s segmentation with a state that had the same label. For each region defined from TF binding sites, we searched for an overlap with a TF binding site detected on that cell type. Finally, for each region defined from open chromatin peaks, we searched for overlap with an open chromatin peak observed in that cell type.

### Comparisons

The VISTA enhancers were downloaded from the Ensembl database. The FANTOM5 enhancers and promoters were downloaded from the FANTOM5 servers [49]. These three sets of regions were remapped from GRCh37 to GRCh38 using liftOver [50]. They were then compared with the Ensembl Regulatory Build using bedtools [51].

### Software tools

The Ensembl eHive framework [52] was used to maximize the efficient use of available compute resources. All the statistical calculations were performed with the WiggleTools library [53].

### Availability and requirements

All Ensembl data and source code are freely available and may be downloaded in their entirety from the Ensembl website [54]. Additionally, the data are available through programmatic Perl, REST interfaces and through the web based Ensembl Biomart. Finally, a track hub [55] contains segmentations, intermediary summary functions and annotations that can be downloaded in bulk. The code used to compute the build is available in script form within the Ensembl Funcgen codebase [56], freely available under an Apache 2 license.

## Abbreviations

ChIP: chromatin immuno-precipitation
IDR: Irreproducibility Discovery Rate
TF: transcription factor
TSS: transcription start site
